# The Effect of Enrichment with Sour-Cherry Extracts on Gluten-Free Snacks Developed by Novel 3D Technologies

**DOI:** 10.3390/antiox12081583

**Published:** 2023-08-09

**Authors:** Evgenia N. Nikolaou, Evangelia D. Karvela, Argyri Papadopoulou, Vaios T. Karathanos

**Affiliations:** 1 Department of Nutrition and Dietetics, Harokopion University of Athens, 70, El. Venizelou Ave., 17671 Athens, Greece; evgenia@hua.gr (E.N.N.); ekarvela@hua.gr (E.D.K.); 2 Department of Food Science and Technology, University of West Attika, Ag. Spyridonos Str., Egaleo, 12243 Athens, Greece; fiqs20022@uniwa.gr

**Keywords:** 3D food printing, gluten-free, enrichment, antioxidant activity, physicochemical properties, drying

## Abstract

Gluten-free formulations (GF) were utilized as food inks enriched with sour-cherry powder (SCP) and lyophilized extract (SCLE), and their physicochemical, rheological, and thermomechanical properties were evaluated with respect to different leavening conditions. Post-printing drying was also assessed in terms of texture, color, and phenolic substances. SCP and SCLE enrichment decreased lightness by 15% and increased red hue by almost 30%, mainly in yeast formulations. SC addition reduced pH by more than 12% for SCP in both leavening conditions and at 10% to 12% for SCLE, depending on leavening agents. The SCLE addition doubled dynamic moduli and complex viscosity magnitudes and increased hardness at 75.7% compared to the control for baking-powder formulations. SC enrichment, compared to the control, increased the phenolic sum to more than 90% (87% SCLE, 96% SCP) in yeast formulations, presenting lower values (almost 70%) for baking powder. Antioxidant activity in 3D-printed snacks was positively influenced by SC incorporation, depending on the drying and leavening treatment. Phenolic content, in terms of total phenolic sum, origin, and composition, possibly impacts the product’s antioxidant activity by depicting antagonistic or synergistic phenomena. Ultimately, 3D printing is feasible for producing functional GF snacks enriched with sour-cherry extracts.

## 1. Introduction

Currently, the snack-food market is displaying a growth potential due to the increased consumption of snack foods associated with the high-pace lifestyle of the public [1,2]. Snack foods present an excellent choice in terms of meeting energy requirements, reducing consumption time, and being vast, readily available options combined with low cost. Moreover, consumers’ constant health awareness has shifted their need to incorporate healthier dietary components in their daily diet, such as bioactive compounds [3,4]. In this context, researchers and the food industry’s attention focuses on the development of functional foods with the incorporation of bioactive components [5].

Another emerging area of high nutritional interest and demand is in gluten-free (GF) products due to the increased prevalence of celiac disease in the population [6,7]. However, gluten-free-based products present difficulties in their production and handling, as the absence of gluten has a significant impact on both dough rheology and quality (mediocre texture, poor color, shelf life, etc.) [7,8,9]. The most adapted approach for improving the rheology and quality of gluten-free doughs is using alternative components that can mimic the viscoelastic properties of the gluten network. Such formulations include mixing gluten-free flours or starch with water, hydrocolloids, emulsifiers, and alternative protein sources [7,8,9,10,11]. Within this context, a combination of hydrocolloids, a starch source, and protein can improve dough structure, enlarge gas retention capacity, and ameliorate gluten-free-bread volume.

One downside of the consumption of GF snacks is their low nutritional value compared with gluten-based formulations. For this reason, it is considered necessary to enrich gluten-free doughs with components that will increase their poor nutritional content with health-promoting substances [12]. A promising way to improve the nutritional value of products, in general, is the incorporation of bioactive compounds from fruits and vegetables, which are rich in antioxidants, vitamins, and fibers [13,14,15]. Recent studies have highlighted the protective role of fruits and vegetables against various diseases, like cancer and cardiovascular diseases, due to the antioxidant action of the bioactive components [16,17]. Sour cherry (*Prunus cerasus* L.) is a worthy source of phytochemical compounds which strongly influence its quality, organoleptic properties, and nutritional value. Specifically, sour cherry is considered rich in phenolic substances, mainly anthocyanins and hydroxycinnamic acids [18,19]. The enrichment of snack foods with sour cherry is innovative and has not been extensively studied, except in the form of encapsulation. Petrovic et al. [20] encapsulated sour-cherry-pomace extract in soy and whey protein carriers in the preparation of cookie dough and showed a significant increase in anthocyanin content, which subsequently caused the change in cookie dough color. Gumul et al. [21] also enriched GF breads with encapsulation of 10% and 20% sour-cherry-pomace in extruded rice flour preparations, successfully increasing the bioactive compound content of GF bread and delaying bread crumb hardness.

To meet these growing demands in snack-food production, emerging food manufacturing technologies can be applied to combine novel ingredients in gluten-free formulations. Three-dimensional printing constitutes a novel technology characterized by a layer-by-layer material deposition based on a pre-designed file in a computer-aided design (CAD) form [22,23]. In the food science sector, 3D food printing (3DFP) enables the creation and production of nutritionally personalized snacks with customizable shapes and complex structures in a simplified food supply chain [24,25].

Evaluation of the physicochemical and mechanical properties of the printing material is a critical factor for ensuring its printability. A material’s viscosity should be both low to favor extrusion and flow during printing and, at the same time, sufficiently high for post-printing structure maintenance [24,26,27]. Within this framework, Radoš et al. [28] concluded that 3D printing of gluten-free powder blends is promising enough to produce personalized snacks of added nutritional value. However, the enrichment of gluten-free products with nutrients can induce physicochemical changes in the final product; thus, extra attention is required during industrial upscaling [29]. In this context, Matas et al. [30] used 3D food printing, incorporating rosehip as a functional ingredient in a gluten-free dough formulation, and studied the physicochemical properties and its bioavailability in a bread matrix. Therefore, a new research-worthy subject emerges regarding the enrichment of gluten-free formulations using novel 3D printing technologies. Within this research, the physicochemical, rheological, and thermomechanical properties of gluten-free dough will provide a knowledge framework linking the material’s properties with the successful 3D printing of structurally intact GF snacks. For this purpose, appropriate screening through diverse types of leavening systems will be performed to suggest the optimal formulation in terms of properties as well as tailored nutritional functionality for use in 3D printing.

Since 3DFP constitutes a novel food manufacturing technology, the impact of extrusion processes on food inks is a subject that requires a fundamental understanding of the occurring phenomena that take place prior to, during, and after the printing process. Concurrently, the 3DFP effect on the structure of printed objects with respect to the post-processing method used has not yet been extensively studied. In most cases, engaging extrusion-based three-dimensional dough printing, a post-processing step, such as baking, steaming, or frying, is required [31]. Post-processing can induce chemical reactions such as the Maillard reaction, protein denaturation, and other physicochemical changes, including moisture reduction and changes in color, volume, and mechanical properties, in the final product [32]. Shape and texture retention, as an after-effect of rapid moisture removal, is a subject worth studying as post-processing induces changes in the size, shape, microstructure, and texture of the final product. In their study, Kim et al. [33] highlighted that the xanthan gum addition to cookie dough can improve its dimensional stability. Zhang et al. [34] investigated the probiotic survival in wheat-flour-based formulations during baking with respect to their surface-to-volume ratio, where an increased probiotic survival rate was established with a well-maintained geometry. Therefore, new knowledge must be generated for designing post-processing methods with respect to novel and improved printing material formulations, providing insight into the physicochemical changes of dough systems.

Within this context, the purpose of this study was the development of gluten-free, starch-based, 3D-printable formulas (dough) enriched with bioactive compounds from sour cherry and the investigation of the effect of enrichment on the physicochemical, rheological, thermal, and mechanical properties of the final product. Moreover, the post-processing of printed dough structures and the effect of two different drying methods (hot air and vacuum drying) on the above-mentioned properties and the total phenolic content and antioxidant capacity of the final product were also evaluated. Dough fortification was performed using two forms of sour-cherry preparations, namely lyophilized extract and lyophilized fruit powder, to evaluate their performance in terms of bioactivity. The effect of the proofing agent type used, yeast and baking powder, on the physicochemical properties of dough and final product before and after fortification was also assessed.

## 2. Materials and Methods

### 2.1. Materials

Corn starch (Merck SA, Athens, Greece), pea protein (Kirpitsas Ingredients, Serres, Greece), xanthan gum from Xanthomonas campestris (Sigma-Aldrich, Steinheim, Germany), frozen sour cherries (Alterra, S.A., Giannitsa, Greece), dry yeast (Sacharomyces cerevisiae, Global Synergy Buying Groups S.A., Thessaloniki, Greece), baking powder (Jotis S.A., Athens, Greece), and all other ingredients used for the preparation of gluten-free dough were locally purchased. Bis-(trimethylsilyl)-trifluoroacetamide (BSTFA), analytical grade ethanol, quercetin, 3-(4-hydroxyphenyl)-1-propanol, homovanillic acid, phloretic acid, oleanolic acid, cinnamic acid, vanillin, p-coumaric acid, chlorogenic acid, catechin, syringic acid, Folin–Ciocalteu reagent, gallic acid, Trolox, DPPH, and TPTZ were obtained from Sigma Chemical Co. (St. Louis, MO, USA). Tyrosol, protocatechuic acid, sinapic acid, o-coumaric acid, and caffeic acid, and epicatechin were purchased from Fluka (Steinheim, Germany); vanillic acid was obtained from Serva (Heidelberg, Germany), while kaempferol, chrysin, naringenin, acacetin, and apigenin was obtained from Extrasynthese (Genay-Cedex, France). Ethanol/water mixture used for the extraction of sour cherry (*Prunus cerasus* L.) was a distillation product from local producers.

### 2.2. Preparation of Gluten-Free Snack Formulations and Sour-Cherry Enrichment Agents

Preliminary experiments were conducted to establish the optimal recipes for each leavening agent and adjust hydrocolloid addition level. Dry ingredients, namely corn starch, pea protein, and salt, were mixed. Regarding the raising agent used, baking powder and freeze-dried sour-cherry powder were added to the dry ingredients, while yeast was first dissolved and stirred in 40 g of warm water and left in the water bath (37 °C, 12–15 min) until its activation was complete. Xanthan gum was pre-mixed with cold water until its dissolution and gel creation. Freeze-dried sour-cherry extract was rehydrated with a small amount of water before adding it to the dry ingredients. The xanthan gum gel, aqueous yeast solution, and olive oil were added, and the mix was kneaded by hand. Recipe ingredients and abbreviations of samples are shown in Table 1. The dough was proofed at 28–30 °C for 15′ for baking powder and 45′ when it contained yeast for the fermentation procedure to be carried out. The amounts of materials used in preparation of corn starch are presented in the table. Sour-cherry-lyophilized powder and lyophilized extract powder were prepared according to methodology described in Nikolaou et al. [17].

### 2.3. Determination of Physicochemical Properties of Dough

#### 2.3.1. Color Analysis of Dough Formulations

Color parameters (L*, a*, b*) were measured before and after proofing to determine the impact of sour-cherry powder or extract addition on color of dough. Prior to color measurement, the instrument (Spectrometer CM-5, Konika Minolta) was calibrated based on the manufacturer’s instructions with black plate, and L* (100 = white, 0 = black), a* (−value = green, +value = red), and b* (−values = blue, +values = yellow) color parameters were measured for each sample in triplicate.

#### 2.3.2. pH, aw, and Moisture Content Determination in Dough Formulations

Before starting the determination of pH, the instrument (SI Analytics Lab 845) was calibrated based on the manufacturer’s instructions. Deionized water up to 100 mL was added to a beaker, along with approximately 2 g of dough, under a magnetic stirrer. Water activity measurements were performed via a water activity meter (Novasina, Lachen SZ, Switzerland, LabSwift-aw) at 25 °C. The moisture content determination in dough was performed according to the official AOAC method; briefly, 2 g of dough sample (approximately 2 g) was weighed and placed in a laboratory oven at 105 °C for 24 h. All measurements were performed in triplicate.

### 2.4. Determination of Thermal Properties of Optimal Dough

Differential Scanning Calorimetry (DSC 6000, Perkin Elmer, Waltham, MA, USA) was used for determination of thermal properties of dough (gelatinization and rearrangement of starch) with respect to the interactions between biopolymers and the powder/lyophilized sour-cherry extract. For each measurement, 8–10 ± 0.1 mg of dough was weighed in aluminum capsules. An empty hermetically sealed aluminum capsule was used as a reference sample. The samples were then hermetically sealed and analyzed (Day 0). Samples were then stored in a conventional refrigerator at 4 °C to investigate starch rearrangement (retrogradation) and reanalyzed on days 5 and 10. On day 0, all samples were isothermally scanned from −40 to 125 °C, and on days 5 and 10, from 10 to 120 °C, with a heating rate of 10 °C/min, wherein the onset (To), peak (Tp), end (Te) temperatures and the enthalpy (ΔH) of gelatinization and retrogradation phenomena were recorded. All measurements were performed in quadruplicate. Calibration of the DSC instrument was performed using an indium standard sample. The processing of the results was carried out using the Pyris v11 software.

### 2.5. Determination of Rheological Properties of Optimal Dough

Rheometer (MCR 102, Anton Paar, GmbH, Graz, Austria), with a parallel plate geometry of 25 mm and a gap set at 2 mm, was used for determination of the rheological properties of the dough samples. An appropriate quantity of the dough sample was placed on the plate; temperature was set at 22 °C. Before measurement, a 5 min relaxation period was accomplished. Amplitude sweeps were performed; shear strain was set at γ = 0.03% within the Linear Viscoelastic Range (L.V.R). Storage modulus (G′), loss modulus (G″), phase angle δ (tan δ, G″/G′), and complex viscosity (η*, Pa·s) were obtained as a function of angular frequency (ω, 0.1–100 rad/s). All measurements were triplicated. Statistical comparisons, where applied, were made at 1 Hz.

### 2.6. 3D Printing of Dough Samples

An extrusion-based 3D food printer (Zmorph.VX.2) was used for all printing experiments. After proofing with baking powder and yeast, processed dough was used as food cartridge (100 mL, food syringe) and placed in the extrusion 3D printing equipment. The parameters of fluidity (thin-thick %) were set in the range of 60–85%, and the height and number of layers, the speed of the nozzle, the speed of printing, the height of the needle, etc., were examined as printing parameters. The optimal conditions were selected for each type of dough. Ten-layered square patterns (30 mm × 30 mm) were 3D-printed, and printability was assessed based on dimensional measurements; after the 3D prints rested for 10 min, Voxelizer 2.0.0 software was used for configuration of the printing parameters as follows: printing speed = 5 mm/s, travelling nozzle speed = 10 mm/s, nozzle diameter: 2 mm, nozzle height = 3 mm. In most cases, the optimal 3D printing conditions were similar; however, in some cases, modifications were made depending on the fluidity of the bioink (dough) and its printability.

### 2.7. Post-Printing Operations

#### 2.7.1. Drying of Printed Dough Samples

Two drying methods, hot air (UOP8 tray drier/Armfield) and vacuum drying (vacutherm VT6025, Heraeus Instruments, Hanau, Germany), were performed for post-processing of 3D-printed dough structures. Drying was carried out at 60 °C for 60 min for both drying methods. The influence of drying methods on the dimensional characteristics of the dough samples was evaluated. Prior to the drying procedure, initial weight and dimensions (length, height, thickness) of the dough samples were measured with a caliper. After 60 min of drying, all samples were weighed to evaluate total water loss and degree of shrinkage as a measure to ensure homogeneous drying of the printed structure.

#### 2.7.2. Texture Analysis of Dough and 3D-Printed Samples

Texture analysis of printed dough samples pre- and post-drying was performed by instrument Texture Analyzer (TA.XT. Plus Stable Micro Systems, Godalming, Surrey, UK). For undried samples, texture profile analysis (TPA) was performed, and one-compression analysis for dried samples. Texture parameters of cohesiveness, gumminess, and springiness were determined for undried samples, and hardness values for both dried and undried samples. For the analysis, measurement parameters were set (speed before compression: 1 mm/s; speed after compression: 10 mm/s; speed during compression: 1 mm/s; and depth of compression: 3 mm/s), and the appropriate probe P/6 was selected. Results were processed with the Exponent Connect v7.0 software. Measurements were performed in triplicate.

### 2.8. Phenolic Compounds Analysis

#### 2.8.1. GC-MS Separation and Identification of Phenolic Compounds

Determination of the initial phenolic compound content, present in sour-cherry powder and extract, was performed by means of GC-MS (Agilent, Wallborn, Germany) coupled with an HP 5973 MS detector, split–splitless injector, and an HP 7683 autosampler. Briefly, an internal standard was added—50 mL of 3-(4-hydroxyphenyl)-1-propanol solution (19.2 μg/mL). Samples derivatization was achieved by 250 mL BSTFA (70 °C for 20 min). Derivatized sample (100 μL) was injected into the GC at a split ratio of 1:20, and MS operation was under electron impact ionization (70 eV) with a scan range of 50–800 Da. An HP-5 MS capillary column (5% phenyl—95% methylsiloxane, 30 m 0.25 mm 250 μm) was used for sample analysis. Helium was used as carrier gas with a flow rate of 0.7 mL/min, and injector and MS detector transfer line temperatures were set at 220 °C and 300 °C, respectively.

#### 2.8.2. Extraction of Bioactive Compounds from the Food Matrix

The impact of drying, as a heat treatment, on total phenolics and the antioxidant capacity of dried samples as a function of sour-cherry enrichment was assessed. Prior to the extraction, dried samples underwent a freeze-drying process in order to remove any residual moisture. The extraction protocol of Conte et al. [35] was adapted with some modifications. Briefly, the dried samples were pulverized for the extraction procedure, and 2 g of powder was weighed into 10 mL volumetric tubes. Then, 4 mL of 37% hydrochloric acid/methanol/water (1/80/19, *v/v/v*) solution was added to the tubes and vortexed for at least 1 min. The extraction took place on a shaking bench in dark conditions for 2 h. The samples were then centrifuged at 3000 rpm for 5 min, and the supernatant was collected (extract). Four more sequential extractions were performed with a 37% hydrochloric acid/methanol/water solution (4 mL each time) with the aforementioned extraction procedure. Subsequently, solvent was evaporated in non-enriched sample extracts by means of rotary vacuum evaporator to a final volume of 4 mL. Extracts were stored at −40 °C until evaluation. GC-MS investigations also took place for the identification of phytochemical compounds present in sour-cherry powder and lyophilized fruit extract, determining the initial bioactive content of the enrichment agents used.

#### 2.8.3. Determination of Total Phenolic Content

Spectrophotometric data were obtained using a Specord 200 (Analytik Jena AG, Jena, Germany) UV–Vis spectrophotometer. Total polyphenol content (T.P.C) of the extracts was determined according to Arnous et al. [36]; for T.P.C, absorbance was read at 750 nm. Gallic acid was used as reference standard, and results were expressed in mg of gallic acid equivalents (GAE) per 100 g d.w.

### 2.9. Determination of Antioxidant Capacity

#### 2.9.1. DPPH Assay

Antioxidant capacity of the dried dough extracts was determined by DPPH spectrophotometric method according to the Arnous et al. [36] protocol, with the appropriate modifications. Absorbance was measured at 515 nm, and antioxidant activity absorbance data were expressed as %ΔA_515_ following Equation (1):

(1) $${\%\Delta A}_{515}=\frac{A_{515}^{t=0}-A_{515}^{t=30}}{A_{515}^{t=0}}\times 100$$

where %ΔA_515_ expresses % decrease in DPPH absorbance, and $A_{515}^{t=0}$ and $A_{515}^{t=30\;}$ express the initial and post 30 min absorbance of DPPH solution and DPPH solution with sample, read at 515 nm.

#### 2.9.2. FRAP (Ferric-Reducing Antioxidant Power) Assay

FRAP spectrophotometric method was also adapted for the determination of antioxidant capacity of dried dough samples according to the protocol of Yilmaz et al. [37], with slight modifications. Absorbance was read at 593 nm, and results were expressed as mM Trolox equivalent (TE) per 100 g of the sample, using the linear regression value calculated from a Trolox calibration curve.

### 2.10. Statistical Analysis

The results were processed with the help of the statistical program SPSS 21 (IBM, Armonk, NY, USA) using One-Way ANOVA and post hoc Tukey test to determine statistically significant differences between samples (*p <* 0.05). Independent samples t-test was performed to evaluate differences among samples subjected to different treatments and processing (leavening conditions, drying procedure).

## 3. Results

### 3.1. Determination of the Physicochemical Properties of Gluten-Free Doughs Enriched with Sour Cherry

The physicochemical properties of doughs enriched with sour-cherry powder and lyophilized extract and treated by different leavening conditions (yeast and baking powder) are presented in Table 2. Color parameters (L*, a*, b*) exhibited statistically significant changes (*p <* 0.05) with respect to the sour-cherry addition. Specifically, the lightness (L*) of the measured samples displayed a significant decrease resulting from the powder and lyophilized extract addition compared with control samples in both leavening conditions (C.B, C.Y). Color parameter a*, which corresponds to red/green hue of the sample, exhibited a significant increase compared with the control formulation in yeast-leavened samples after fortification with sour-cherry powder/lyophilized extract. For the b* parameter (yellow/blue hue), a significant decrease (*p <* 0.05) with the addition of sour-cherry powder/lyophilized extract was also observed in comparison with control samples for both raising conditions.

According to Table 2, no significant changes were observed regarding moisture content as a result of sour-cherry powder and lyophilized extract addition, while significantly (*p <* 0.05) lower moisture content values were observed for yeast formulations compared to those of baking powder. In the same context, water activity values also presented lower values in yeast formulations compared to baking powder, while no significant differences were established as a function of sour-cherry enrichment. Measured pH (on enriched doughs) showed significant differences (*p <* 0.05) among different proofing conditions, with pH ranging from 5.7–6.5 in yeast-leavened samples and 7.1–8.2 for baking-powder-leavened formulations. A significant pH decrease (*p <* 0.05) was observed in both leavening conditions with the addition of sour-cherry powder/lyophilized extract (Table 2), with respect to the control sample, related to the acidic nature of sour cherry.

### 3.2. Determination of Rheological Properties of Optimal Dough

**Figure 1:**
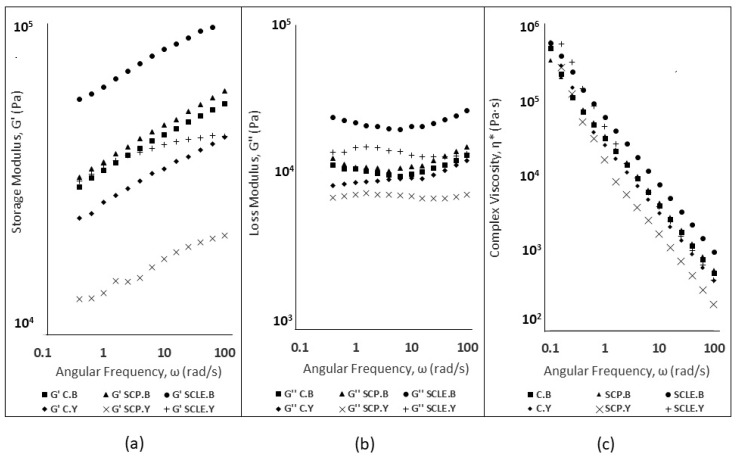
(**a**) Storage Modulus (G′, Pa) for Baking-powder- and Yeast-leavened Samples; (**b**) Loss Modulus (G″, Pa) for Baking-powder- and Yeast-leavened Samples; and (**c**) Complex Viscosity (η*, Pa·s) for Baking-powder- and Yeast-leavened Doughs. C: Control Formulation; Y: Gluten-free Formulations with Dry Yeast; B: Gluten-free Formulations with Chemical Reagent Baking Powder; SCP: Sour-cherry Fruit Powder; SCLE: Sour-cherry Extract in Powder Form.

The viscoelastic parameters of gluten-free dough samples, measured at a frequency of 1Hz, are displayed in Table 3, and their mechanical spectra as a function of angular frequency are displayed in Figure 1. As can be observed from Table 3 and Figure 1, the values of the storage modulus were dominant over the loss modulus values of the (G′ > G″) in all samples, suggesting a more solid, elastic-like dough behavior. This great dependence of dynamic moduli (G′ and G″) on angular frequency is also displayed in phase angle δ values (0.1 < tan δ < 1) (Table 3), confirming weak gel behavior, as has previously been suggested in studied starch-based gluten-free systems [38,39]. Doughs with lower tan δ values have been generally related to a strengthening effect on dough structure compared with high tan δ values [39]. This behavior was established in the case of baking-powder-leavened formulations in comparison with yeast-leavened doughs, suggesting a softer texture of the latter, which was also displayed by the lower values of dynamic moduli of doughs prepared with yeast.

Storage modulus (G′) and loss modulus (G″) dependence on angular frequency (ω) is shown in Figure 1a,b. In baking-powder-leavened samples, both moduli displayed shear-dependent behavior, increasing with angular frequency (range 0.1 to 100 rad/s), which has been reported for gluten-free dough systems [40], while less shear dependency was established for yeast-leavened samples. The magnitudes of dynamic moduli (G′, G″) for SCLE were significantly higher compared to the control formulation for baking-powder formulations, suggesting that the incorporation of sour-cherry extract has a structure-promoting ability in GF doughs. An increase of G′, G″ values was also established for SCLE yeast formulations, but it was not regarded as significant. The incorporation of sour-cherry powder (SCP) did not seem to significantly modify the viscoelastic characteristics in both leavening conditions with respect to the control sample. However, a significant decreasing effect on dynamic moduli was established for yeast formulations compared to SCLE addition, suggesting reduced elasticity. This could possibly be attributed to the acidification effect of SCP on the dough’s structure which is associated with structure weakening of the dough’s matrix and a more liquid-like behavior [41].

Complex viscosity (η*, Pa·s) curves of dough samples for both leavening conditions are shown in Figure 1c. A decreasing trend of complex viscosity values was observed in all studied samples, being consistent with the non-Newtonian behavior (pseudoplastic) that has been reported in gluten-free dough systems [42]. On a general note, baking-powder-leavened doughs displayed higher complex viscosity values compared to yeast formulations, which was associated with a greater difficulty in the former samples to be extruded through the nozzle. The SCLE addition doubled the complex viscosity magnitude in baking-powder formulations compared to the control, while the SCP addition did not seem to induce any changes in η* values. For yeast formulations, the SCP addition displayed decreased complex viscosity values, compared to SCLE, due to pH decrease that can induce changes in gluten-free doughs, decreasing viscosity and weakening the dough’s structure [41]. The lyophilized extract addition in baking-powder-leavened formulations was found to induce an increase in complex viscosity values, while no significant differences in η* (Pa·s) were established from the addition of sour-cherry powder or lyophilized extract in dough, reflecting the ease in extrusion for these three samples (C.Y, SCP.Y, SCLE.Y) during 3D printing.

### 3.3. Determination of Thermal Properties of Optimal Dough

Regarding the investigation of the thermal properties in gluten-free dough formulations, the determination of ΔH values corresponds to the energy released during the melting of the starch crystalline region and, more specifically, the double-helical structures of amylopectin crystalline regions [38]. This form of native corn starch is usually displayed in a thermograph as an endothermic transition appearing at 60–73 °C in an abundance of available water [43]. However, the partial restriction of this transition can be observed when the available water is limited due to the presence of hydrocolloids such as xanthan gum, which can retard swelling of the starch granule. This results in the onset–endset gelatinization being postponed at a higher temperature range, as was observed for all dough samples, which displayed the typical gelatinization endotherm at approximately 72 °C (day 0, Table 4). In general, the addition of sour cherry powder and lyophilized extract did not seem to induce any statistically important difference in the onset–endset and melting enthalpy of the studied samples with respect to the control formulation. However, differences (*p <* 0.05) were observed between the two different raising conditions used, with the yeast formulations exhibiting generally lower temperature range and peak for gelatinization, possibly due to pH variations of doughs.

The staling properties of the prepared gluten-free snacks containing different types of sour-cherry-enrichment agents were investigated with respect to the starch retrogradation phenomenon of samples stored at 4 °C for 5 and 10 days; results are displayed in Table 4. The thermal transition observed for aged samples corresponding to the retrogradation endotherm produced non-significant results regarding the impact of enrichment on the retrogradation phenomenon.

### 3.4. Texture Analysis of Dough and Post-Printed Operations in 3D-Printed Samples

Texture profile analysis (TPA) results of gluten-free samples as a function of enrichment of sour-cherry bioactive compounds and leavening conditions are shown in Table 5. As can be observed, hardness in both leavening conditions was increased with the addition of sour-cherry lyophilized extract. These results are in line with the obtained rheological data, where SCLE addition was shown to increase the dynamic moduli compared to control formulations.

**Figure 2:**
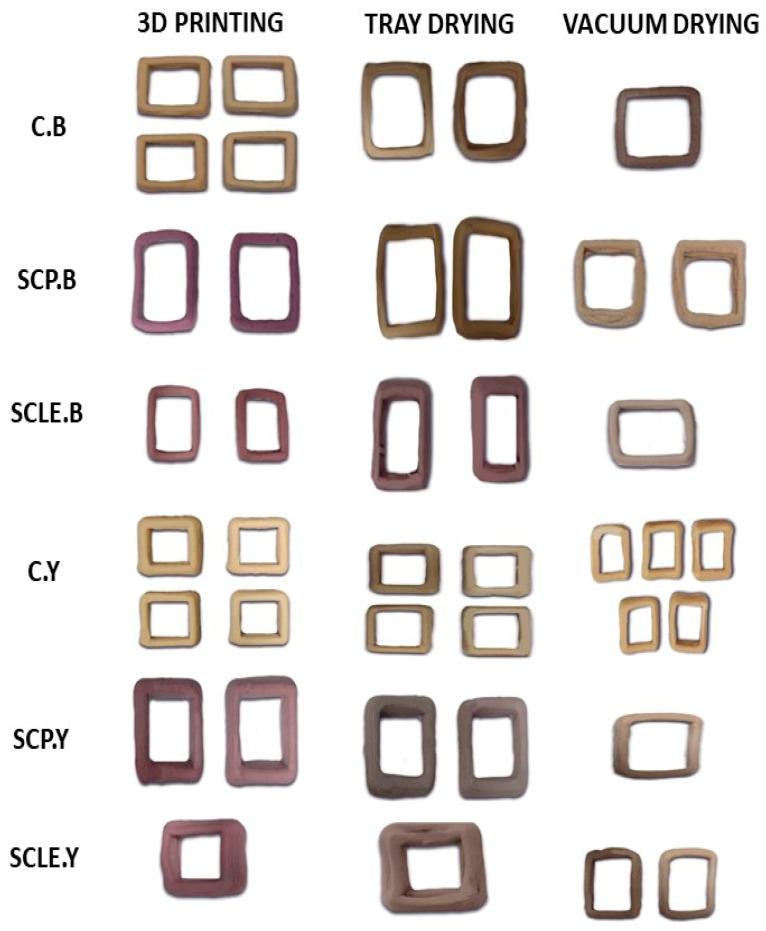
3D-printed food constructs, treated with different drying methods (tray and vacuum drying). C: Control Formulation; Y: Gluten-free Formulations with Dry Yeast; B: Gluten-free Formulations with Chemical Reagent Baking Powder; SCP: Sour-cherry Fruit Powder; SCLE: Sour-cherry Extract in Powder Form.

With respect to the different raising conditions used, measured texture parameters displayed significantly (*p <* 0.05) lower values for yeast-leavened samples compared to baking-powder formulations. Εxtrusion behavior was facilitated for samples prepared with yeast rather than baking-powder formulations, as a better printing flow was achieved, resulting in more spreadable dough structures (Figure 2). Cohesiveness is a texture parameter directly associated with the intrinsic structural resistance within a food matrix and the sample’s ability to stick to itself. Dough’s cohesiveness was found to decrease with the addition of sour-cherry powder/lyophilized extract in baking-powder formulations, while the opposite was observed for yeast-formulated dough samples, which could be related to pH differences. In general, baking-powder samples were found to be more cohesive than yeast-leavened ones, which could be potentially linked to a better binding ability amongst sequentially deposited dough layers for baking-powder samples.

Post-printing, samples were subjected to drying, and significant changes regarding dimensional shrinkage and color hue of the 3D-printed structures were observed (Figure 2). The impact of different drying methods on color parameters is shown in Table 6. L* parameter for vacuum-dried samples was found to be significantly increased compared with tray-dried samples. Lightness was also significantly decreased with enrichment with sour-cherry powder/lyophilized extract. Between the two different raising conditions, yeast-leavened samples exhibit significantly higher L* values (*p <* 0.05) as opposed to baking powder.

Regarding texture as a function of drying, it was observed that hardness for samples processed in a tray dryer was significantly higher (*p <* 0.05) in comparison with vacuum drying. This was anticipated due to the higher water loss induced by the hot-air stream drying. For yeast samples, no significant differences were observed in both drying conditions as a result of the sour-cherry addition. However, in baking-powder samples, hardness was found significantly (*p <* 0.05) increased in both drying processes when supplementation was carried out through sour-cherry lyophilized extract.

### 3.5. Phenolic Compound Analysis

#### 3.5.1. GC-MS Separation and Identification of Phenolic Compounds

GC-MS method was used to determine phenolic substances of enrichment matrices (sour-cherry powder and lyophilized extract). Vanillin, cinnamic, protocatechuic, gallic, ferulic, caffeic, chrysin, epicatechin, catechin, chlorogenic, and quercetin were identified in both matrices in different concentrations, presenting substantially higher percentages for sour-cherry powder. Tyrosol, p-OH benzoic, phloretic, vallinic, syringic, sinapic, oleanolic, ursolic acids, kampherol, and naringenic were detected only in sour-cherry powder, suggesting the poor or not-detectable presence of these phenolics in the extract, possibly due to the extraction process.

#### 3.5.2. Determination of Total Phenolic Content

**Figure 3:**
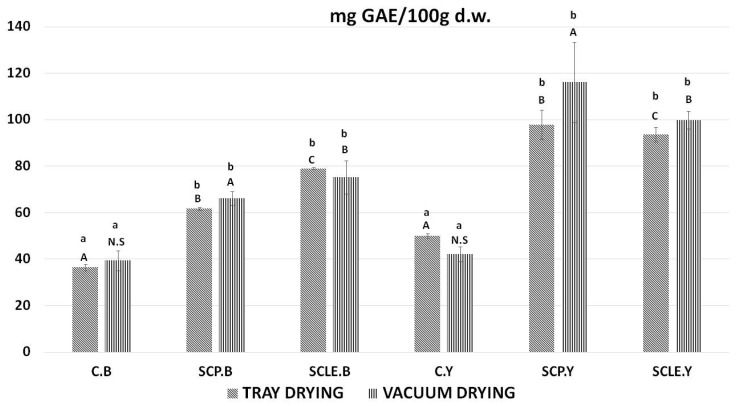
Total polyphenol content expressed as mg GAE/100 g d.w. Displayed data are expressed as mean values of three replications ± standard deviation. Values in the same column not sharing (i) lowercase superscript letters suggest significant differences (*p <* 0.05) amongst samples at a confidence level of 95%; (ii) uppercase subscript letters suggest significant differences (*p <* 0.05) amongst samples treated with different raising conditions at a confidence level of 95%. N.S: not significant differences (*p <* 0.05) amongst samples treated with different raising conditions (*p >* 0.05). C: Control Formulation; Y: Gluten-free Formulations with Dry Yeast; B: Gluten-free Formulations with Chemical Reagent Baking Powder; SCP: Sour-cherry Fruit Powder; SCLE: Sour-cherry Extract in Powder Form; TD: Tray-Drying Process; VD: Vacuum-Drying Process.

In Figure 3, the bioactive content, in terms of total polyphenol content, of 3D-printed doughs as a function of the drying process used is presented. For baking-powder formulations, the addition of sour-cherry powder and lyophilized extract was shown to increase total polyphenol content in tray-drying treatment by 25.37 ± 0.63 (SCP), 33.88 ± 0.31 (SCLE) mg GAE/100 d.w. and in vacuum-drying treatment 26.82 ± 1.31, 35.89 ± 1.11 mg GAE/100 d.w., respectively. For dry yeast formulations, the respective increased values were 47.88 ± 0.93 (SCP), 43.59 ± 1.25 (SCLE) mg GAE/100 d.w. in the tray-drying treatment and 71.65 ± 1.28 mg GAE/100 d.w. and 55.36 ± 1.83 in the vacuum-drying treatment. Therefore, the incorporation of sour-cherry powder or lyophilized extract increased polyphenol content compared to the control formulation in both drying conditions, being more pronounced in SCLE for baking-powder-based formulations and SCP for yeast formulations, respectively. The total polyphenol content found in the investigated dried snacks was found to differ significantly among different raising conditions, with yeast-leavened samples exhibiting higher TPC than baking-powder formulations for both drying treatments.

#### 3.5.3. DPPH Assay

**Figure 4:**
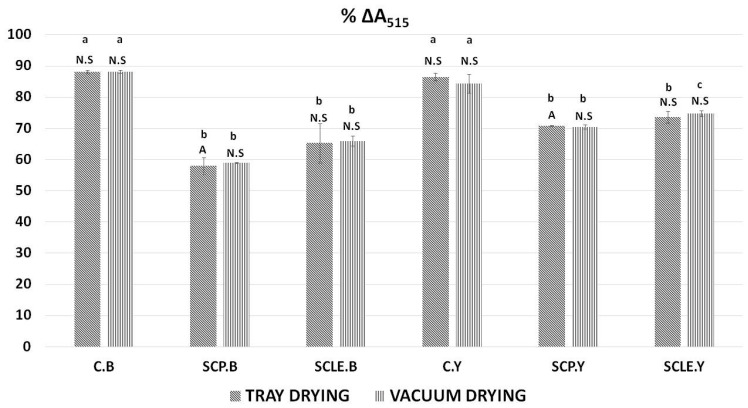
Antioxidant activity by DPPH assay expressed as % ΔA_515_. Displayed data are expressed as mean values of three replications ± standard deviation. Values in the same column not sharing (i) lowercase superscript letters suggest significant differences (*p <* 0.05) amongst samples at a confidence level of 95%; (ii) uppercase subscript letters suggest significant differences (*p <* 0.05) amongst samples treated with different raising conditions at a confidence level of 95%. N.S: not significant differences (*p <* 0.05) amongst samples treated with different raising conditions (*p >* 0.05). C: Control Formulation; Y: Gluten-free Formulations with Dry Yeast; B: Gluten-free Formulations with Chemical Reagent Baking Powder; SCP: Sour-cherry Fruit Powder; SCLE: Sour-cherry Extract in Powder Form; TD: Tray-Drying Process; VD: Vacuum-Drying Process.

The decrease in % ΔA_515_ was used to determine the antioxidant activity of 3D-printed gluten-free samples as a function of sour-cherry addition and different drying treatments. Results can be observed in Figure 4. As was expected, control samples (C.B, C.Y) did not exhibit significant differences among the different drying treatments and raising conditions used. However, a comparison between the control formulations, without the addition of sour cherry with the enriched formulations, indicated significant differences (*p <* 0.05) for both raising conditions being more pronounced in yeast formulations. The type of raising agent had no significant impact on the % decrease of % ΔA_515_, except for the sour-cherry-powder-enriched sample processed with tray-drying (SCP.B.TD) that differentiated significantly (*p <* 0.05) between the two conditions.

#### 3.5.4. FRAP Assay

**Figure 5:**
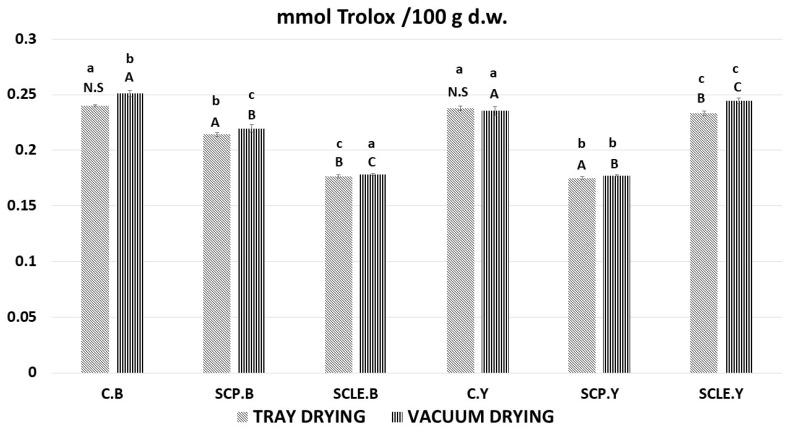
Antioxidant content by FRAP assay, expressed as mmol Trolox/100 g d.w. Displayed data are expressed as mean values of three replications ± standard deviation. Values in the same column not sharing (i) lowercase superscript letters suggest significant differences (*p <* 0.05) amongst samples at a confidence level of 95%; (ii) uppercase subscript letters suggest significant differences (*p <* 0.05) amongst samples treated with different raising conditions at a confidence level of 95%. N.S: not significant differences (*p <* 0.05) amongst samples treated with different raising conditions (*p >* 0.05). C: Control Formulation; Y: Gluten-free Formulations with Dry Yeast; B: Gluten-free Formulations with Chemical Reagent Baking Powder; SCP: Sour-cherry Fruit Powder; SCLE: Sour-cherry Extract in Powder Form; TD: Tray-Drying Process; VD: Vacuum-Drying Process.

The results of the FRAP assay, as a function of different means of enrichment with sour-cherry bioactive substances and different raising conditions, are presented in Figure 5. All samples displayed ferric-reducing capacity; however, results were contradictory to DPPH and TPC findings since the capacity seems to decrease with increasing added antioxidant content. Although increased percentages of antioxidant capacity would be expected due to enrichment, further study is required that focuses on the mechanism of the leavening mode and the synergism–competition of antioxidants from different sources (virgin olive oil extract), since a quite complex food matrix is involved, suggesting the occurrence of more composite physicochemical phenomena such as Maillard reaction.

## 4. Discussion

The fortification of GF products with bioactive compounds derived from fruits such as sour cherry is a promising way to enhance the nutritional profile of products because of their well-documented antioxidant action. GC-MS analysis performed on sour-cherry extract and powder characterized their rich bioactive compound profile, suggesting that SCP addition, compared to SCLE, is a highly potent means for the fortification of snack formulations. A positive impact on the quality parameters as a function of rheological, mechanical, and physicochemical properties and bioactivity content was established for both fortification methods. This highlights the possible health-promoting profile of the prepared gluten-free products. Moreover, the application of 3D printing combined with post-print-drying processing (for the development of enriched GF snacks with sour cherries) is a subject that has not yet been explored in the existing literature and is worth studying. The incorporation of SCP and SCLE on the physicochemical properties of dough (color parameters, pH, moisture content, aw) showed significant changes that were dependent on the leavening conditions used (baking powder, yeast). Enriched doughs presented a pH decrease compared to the control formulation under both leavening conditions. This pH decrease was expected due to naturally presented organic acids present in sour cherry’s phytochemical extracts and fruit, which can reduce the pH of starch–water suspensions [44]. The observed changes in color were attributed to the SCP and SCLE color intensity, which is associated with their rich anthocyanin content. Similar results were obtained by Petrovic et al., where encapsulated sour-cherry pulp was used in whey-protein carriers for biscuit enrichment [20]. Variations in color parameters between the enriched doughs as a function of the leavening agent are also displayed. As has been reported in the literature, anthocyanin color is highly dependent on its medium, displaying red tones in acidic conditions and blueish tones in alkaline media [45,46].

Moisture content and water activity did not present any differences due to sour-cherry fortifications, possibly because of the presence of xanthan gum in GF dough, which, even at relatively low concentrations, affects the rheology of dough, increasing its viscosity and retaining high amounts of water. However, their values varied as a function of leavening conditions, with yeast presenting lower values for both moisture content and water activity because yeast development in the dough matrix increases its protein content and thus requires a greater amount of available water.

The rheological and thermomechanical properties of the enriched and control doughs were evaluated with respect to their 3D printing performance and interactions between food components. The determination of the rheological characteristics of gluten-free doughs, focusing on their viscoelastic properties, can provide insight into the interactions amongst ingredients as well as the manufacturing process that can cause changes in the rheological response of dough during oscillation. A predominance of the elastic behavior over the viscous behavior of dough was observed for all samples. This behavior is associated with the printability factor of doughs as a 3D food cartridge and is indicative of the stability of the printed structure after deposition, as it reflects elasticity sufficiency and mechanical stability for 3D printing [30]. Another critical factor associated with the printing behavior in extrusion-based 3D food printing is the viscosity of the material. All the samples exhibited shear thinning behavior (pseudoplasticity) with increasing angular frequency (ω, rad/s). Pseudoplasticity is considered essential in 3D printing applications, as it ensures the adequate fluidity of the printing material during extrusion [27]. The investigation of the thermal properties in terms of starch gelatinization as a function of sour-cherry enrichment did not produce any significant results. However, differences were observed in the gelatinization temperature range between the different leavening conditions, which could be attributed to the pH differences among samples due to the interaction of sour-cherry phytochemicals with available water molecules.

The complexity of the starch retrogradation phenomenon in enriched doughs requires further research with analytical methods such as FTIR, focusing on the occurrence of starch–polyphenol interactions. Texture profile analysis performed on dough samples highlighted the differences between the two leavening methods, with yeast formulations presenting lower hardness, cohesiveness, and gumminess values than baking powder. This is indicative of the generally softer texture that yeast-leavened samples exhibited during extrusion through the 3D printing nozzle. This phenomenon could also be associated with the decreased water activity of samples, as well as the increased protein content and fermentation process reactions resulting from yeast development, which also contribute to dough flexibility [15]. Overall, the extrusion behavior was facilitated for samples prepared with yeast rather than baking-powder formulations, as a better printing flow was achieved, resulting in more spreadable dough structures. Similar results were obtained by the authors when supplementing wheat-based bakery products with grape phytochemical-rich formulations in the form of powder and lyophilized extract [17].

The fabricated 3D-printed GF constructs were further subjected to different drying processes (tray and vacuum drying) to evaluate the impact of the process on the bioactivity of the final product. The total polyphenol content increased, and %ΔA_515_ decreased compared to the control formulation because of the fortification process in both the leavening conditions and drying processes used. These findings suggest that the sour-cherry addition to the GF dough matrix significantly improves the bioactive content of the 3D-printed dough and that drying of 3D-printed GF snacks performed at 60 °C for 1 h is potent for maintaining this content. In contrast to the above-discussed results, the antioxidant capacity (FRAP assay) showed a decreasing trend with increasing antioxidant content, which is possibly attributed to synergistic–antagonistic phenomena between antioxidants. Similar studies have highlighted this antagonistic–synergistic effect among different antioxidants in complex mixtures, which has been attributed to (i) antioxidant polymerization due to oxidation, (ii) regeneration of compounds with decreased antioxidant capacity by a more efficient one, and (iii) complex and adduct formation amongst bioactive compounds [47,48]. Moreover, sugars and organic acids have been reported to possess an antagonistic–synergistic role in the total antioxidant activity of intrinsic polyphenols, which is dependent on the concentration and polyphenol type [49].

## 5. Conclusions

Supplementation of gluten-free formulations with sour-cherry powder and extract in a lyophilized powder form impacted the attributes of several qualities of the developed GF snack. The extent of this effect was highly influenced by the conditions used during preparation. Sour-cherry enrichment reduced pH values compared to the control by more than 12% in SCP samples in both leavening conditions and by 10% to 12% for SCLE, depending on the leavening agent. Color parameters were significantly influenced by the addition of sour-cherry due to pH changes resulting from the acidic nature of the sour-cherry fruit itself, as well as the proofing process. Decreased lightness values by 15% and increased red hue values (almost 30%) were obtained from SCP and SCLE enrichment, mainly in yeast formulations. The SCLE addition in baking-powder samples induced significant changes in the dynamic moduli, doubling their magnitudes compared to the control, which was observed in the increased hardness values (by 75.7%). With respect to thermal properties, no significant differences were established in gelatinization for both sour-cherry forms of addition, except for different raising conditions where a decreased temperature range for gelatinization was observed for yeast-treated dough formulations compared to baking-powder formulations for all samples. Post-printing drying of 3D-printed dough structures revealed changes in color parameters due to sour-cherry supplementation; thus, differentiation among samples was also observed as a function of different drying methods. Hardness yielded increased values in the tray-drying process as opposed to vacuum drying. Spectrophotometric analyses suggested that the incorporation of sour-cherry powder and extract in the form of lyophilized powder increased the total phenolic content by 90% (87% SCLE, 96% SCP) in yeast formulations and almost 70% in baking powder compared to the control. Antioxidant activity was also positively influenced by the addition of sour-cherry compared to that of the control samples under both raising conditions. The presence of phenolic compounds with different origins in a complex food system, such as a gluten-free snack, in addition to the influence of the low heat treatment (60 °C) and 3D printing process, need to be studied further in terms of the antioxidant properties and phenolic profile of the final product. The process of fortification of starch-based gluten-free snacks with sour-cherry bioactive compounds using 3D food printing technology, as described in this study, is a promising way to produce functional foods with potentially health-promoting effects.

## Figures and Tables

**Table 1:** Composition of prepared gluten-free dough formulations with or without the addition of sour-cherry powder or lyophilized extract.

Ingredients (g)	C.Y	SCP.Y	SCLE.Y	C.B	SCP.B	SCLE.B
Raising Agent	Yeast			Baking Powder		
Corn Starch	63	63	63	63	63	63
Pea Protein	30	30	30	30	30	30
Xanthan Gum	0.5	0.5	0.5	0.5	0.5	0.5
Sour-Cherry Addition	-	5	5	-	5	5
Dry Yeast	2	2	2	-	-	-
Baking Powder	-	-	-	4	4	4
Water	110	110	110	110	110	110
Sugar	4	4	4	-	-	-
Salt	1	1	1	1	1	1
Olive Oil	8	8	8	8	8	8

C: Control Formulation; Y: Gluten-free Formulations with Dry Yeast; B: Gluten-free Formulations with Chemical Reagent Baking Powder; SCP: Sour-cherry Fruit Powder; SCLE: Sour-cherry Extract in Powder Form.

**Table 2:** Physicochemical parameters of gluten-free doughs with yeast and baking powder as raising agents.

Samples	Color Parameters			Moisture (%) d.w.	Physicochemical Properties	
	L*	a*	b*		aw	pH
	Raising Agent: Baking Powder					
C.B	76.2 ±$\;0.2{\;}_{\mathrm{NS}}^{a}$	5.3 ± $0.1{\;}_{\mathrm{NS}}^{\mathrm{ns}}$	23.4 ± $0.2{\;}_{A}^{a}$	53.32 ± $0.26{\;}_{A}^{\mathrm{ns}}$	0.969 ± ${0.001\;}_{A}^{\mathrm{ns}}$	8.2 ± $0.3{\;}_{A}^{a}$
SCP.B	62.1 ± $0.5{\;}_{A}^{b}$	5.5 ± $1.3{\;}_{\mathrm{NS}}^{\mathrm{ns}}$	12.2 ±$\;0.3{\;}_{\mathrm{NS}}^{b}$	52.99 ± $0.06{\;}_{\mathrm{NS}}^{\mathrm{ns}}$	0.965 ± $0.002{\;}_{B}^{\mathrm{ns}}$	7.1 ± $0.0{\;}_{B}^{b}$
SCLE.B	60.6 ± $2.0{\;}_{\mathrm{NS}}^{b}$	3.4 ± $1.2{\;}_{A}^{\mathrm{ns}}$	12.1 ± $1.0{\;}_{B}^{b}$	54.08 ±$\;0.20{\;}_{B}^{\mathrm{ns}}$	0.962 ± $0.001{\;}_{C}^{\mathrm{ns}}$	7.2 ± $0.1{\;}_{C}^{b}$
	Raising Agent: Dry Yeast					
C.Y	75.7 ±$\;0.3{\;}_{\mathrm{NS}}^{a}$	5.0 ± $0.1{\;}_{\mathrm{NS}}^{a}$	22.8 ± $0.2{\;}_{A}^{a}$	51.70 ± $0.15{\;}_{A}^{\mathrm{ns}}$	0.934 ± $0.002{\;}_{A}^{\mathrm{ns}}$	6.5 ± $0.1{\;}_{A}^{a}$
SCP.Y	65.0 ± $0.6{\;}_{A}^{b}$	6.4 ± $0.4{\;}_{\mathrm{NS}}^{b}$	12.8 ±$\;0.4{\;}_{\mathrm{NS}}^{b}$	51.84 ± $0.43{\;}_{\mathrm{NS}}^{\mathrm{ns}}$	0.938 ± $0.001{\;}_{B}^{\mathrm{ns}}$	5.7 ± $0.1{\;}_{B}^{b}$
SCLE.Y	63.8 ± ${0.3\;}_{\mathrm{NS}}^{b}$	6.7 ± $0.0{\;}_{A}^{b}$	14.9 ± $0.2{\;}_{B}^{c}$	51.52± $0.19{\;}_{B}^{\mathrm{ns}}$	0.934 ± $0.001_{\;A,B}^{\;\mathrm{ns}}\;$	5.8 ± $0.8{\;}_{C}^{b}$

Displayed data are expressed as mean values of three replications ± standard deviation. Values in the same column not sharing (i) lowercase superscript letters suggest significant differences (*p <* 0.05) amongst samples at a confidence level of 95%; ns: not significant differences amongst samples (*p >* 0.05); (ii) uppercase subscript letters suggest significant differences (*p <* 0.05) amongst samples treated with different raising conditions at a confidence level of 95%. NS: Not significant treatment with different raising conditions (*p >* 0.05). C: Control Formulation; Y: Gluten-free Formulations with Dry Yeast; B: Gluten-free Formulations with Chemical Reagent Baking Powder; SCP: Sour -cherry Fruit Powder; SCLE: Sour-cherry Extract in Powder Form.

**Table 3:** Viscoelastic parameters of gluten-free dough samples measured at 1 Hz.

Samples	G′ (Pa)	G″ (Pa)	tanδ	η* (Pa·s)
Raising Agent: Baking Powder				
C.B	42,442 ± $413{\;}_{A}^{a}$	9853 ± $1612{\;}_{\mathrm{NS}}^{a}$	0.232 ± $0.01{\;}_{A}^{\mathrm{ns}}$	6906 ± $893{\;}_{A}^{a}$
SCP.B	45,509 ± $7347{\;}_{B}^{a}$	11,093 ± $1534{\;}_{\mathrm{NS}}^{a}$	0.245 ± $0.01{\;}_{B}^{\mathrm{ns}}$	7442 ± $1189{\;}_{B}^{a}$
SCLE.B	81,064 ± $14,593{\;}_{C}^{b}$	20,014 ± $2813{\;}_{\mathrm{NS}}^{b}$	0.248 ± $0.01{\;}_{C}^{\mathrm{ns}}$	13,234 ± $2352{\;}_{\mathrm{NS}}^{b}$
Raising Agent: Dry Yeast				
C.Y	31,433 ± $3628{\;}_{A}^{a,b}$	9005 ± $771{\;}_{\mathrm{NS}}^{a,b}$	0.287 ± $0.01{\;}_{A}^{a}$	5182 ± $585{\;}_{A}^{a,b}$
SCP.Y	16,526 ± $2663{\;}_{B}^{a}$	7349 ± $418{\;}_{\mathrm{NS}}^{a}$	0.452 ± $0.07{\;}_{B}^{b}$	2869 ± $396{\;}_{B}^{a}$
SCLE.Y	40,107 ± $12,980{\;}_{C}^{b}$	14,299 ± $4406{\;}_{\mathrm{NS}}^{b}$	0.358 ± $0.01{\;}_{C}^{a}$	6749 ± $2172{\;}_{C}^{b}$

Displayed data are expressed as mean values of three replications ± standard deviation. Values in the same column not sharing (i) lowercase superscript letters suggest significant differences (*p <* 0.05) amongst samples at a confidence level of 95%; ns: not significant differences amongst samples (*p >* 0.05); (ii) uppercase subscript letters suggest significant differences (*p <* 0.05) amongst samples treated with different raising conditions at a confidence level of 95%. NS: Not significant treatment with different raising conditions (*p >* 0.05). C: Control Formulation; Y: Gluten-free Formulations with Dry Yeast; B: Gluten-free Formulations with Chemical Reagent Baking Powder; SCP: Sour-cherry Fruit Powder; SCLE: Sour-cherry Extract in Powder Form.

**Table 4:** Gelatinization and retrogradation properties of enriched and control dough samples stored at 4 °C. Thermal parameters displayed: onset temperature (To), conclusion temperature (Tend), peak temperature (Tpeak), and gelatinization and retrogradation enthalpy for 0 and 5 and 10 days, respectively (ΔH).

Raising Agent: Baking Powder						Raising Agent: Dry Yeast					
Sample	Storage (Days)	Ton (°C)	Tend (°C)	Tpeak (°C)	ΔH (j/g)	Sample	Storage (Days)	Ton (°C)	Tend (°C)	Tpeak (°C)	ΔH (j/g)
C.B	0	73.9 ± $0.5{\;}_{A}^{\mathrm{ns}}$	92.1 ± $2.6{\;}_{A}^{\mathrm{ns}}$	78.7 ± $0.6{\;}_{A}^{\mathrm{ns}}$	3.0 ± $\;0.6{\;}_{\mathrm{NS}}^{\mathrm{ns}}$	C.Y	0	71.7 ± $0.7{\;}_{A}^{\mathrm{ns}}$	84.9 ± $0.4{\;}_{A}^{\mathrm{ns}}$	76.4 ± $0.4{\;}_{A}^{\alpha }$	3.5 ± ${1.8\;}_{\mathrm{NS}}^{\mathrm{ns}}$
	5	43.5 ±$\;0.75{\;}_{\mathrm{NS}}^{\mathrm{ns}}$	69.4 ± $2.7{\;}_{\mathrm{NS}}^{\mathrm{ns}}$	55.8 ± $\;0.9{\;}_{\mathrm{NS}}^{\mathrm{ns}}$	2.8 ± $\;0.3{\;}_{\mathrm{NS}}^{\mathrm{ns}}$		5	48.7 ± $\;1.7{\;}_{\mathrm{NS}}^{\mathrm{ns}}$	63.6 ± $1.1_{\;\mathrm{NS}}^{\;\mathrm{ns}}$	55.6 ± $1.2_{\;\mathrm{NS}}^{\;\mathrm{ns}}$	2.8 ± $0.6_{\;\mathrm{NS}}^{\;\mathrm{ns}}$
	10	45.1 ± $\;0.4{\;}_{\mathrm{NS}}^{\mathrm{ns}}$	66.1 ± $1.3{\;}_{\mathrm{NS}}^{\mathrm{ns}}$	56.2 ± $\;0.8{\;}_{\mathrm{NS}}^{\mathrm{ns}}$	2.0 ± $\;0.4{\;}_{\mathrm{NS}}^{\mathrm{ns}}$		10	45.4 ± $\;1.9{\;}_{\mathrm{NS}}^{\mathrm{ns}}$	62.2 ± $1.2_{\;\mathrm{NS}}^{\;\mathrm{ns}}$	54.0 ± $1.7_{\;\mathrm{NS}}^{\;\mathrm{ns}}$	1.4 ± $0.3_{\;\mathrm{NS}}^{\;\mathrm{ns}}$
SCP.B	0	74.3 ± ${0.3\;}_{B}^{\mathrm{ns}}$	86.4 ± ${0.8\;}_{B}^{\mathrm{ns}}$	78.9 ± ${0.2\;}_{B}^{\mathrm{ns}}$	3.2 ± ${0.4\;}_{\mathrm{NS}}^{\mathrm{ns}}$	SCP.Y	0	72.4 ± ${0.3\;}_{B}^{\mathrm{ns}}$	84.0 ± ${0.4\;}_{B}^{\mathrm{ns}}$	76.9 ± ${0.2\;}_{Β}^{\alpha ,b}$	3.3 ± ${0.5\;}_{\mathrm{NS}}^{\mathrm{ns}}$
	5	43.7 ± ${1.1\;}_{\mathrm{NS}}^{\mathrm{ns}}$	68.0 ± ${0.6\;}_{\mathrm{NS}}^{\mathrm{ns}}$	56.1 ± ${0.4\;}_{\mathrm{NS}}^{\mathrm{ns}}$	1.8 ± ${0.4\;}_{\mathrm{NS}}^{\mathrm{ns}}$		5	44.2 ±${1.7\;}_{\mathrm{NS}}^{\mathrm{ns}}$	66.0 ± ${0.8\;}_{\mathrm{NS}}^{\mathrm{ns}}$	54.2 ±${0.5\;}_{\mathrm{NS}}^{\mathrm{ns}}$	1.4 ± ${1.1\;}_{\mathrm{NS}}^{\mathrm{ns}}$
	10	43.3 ± ${0.4\;}_{\mathrm{NS}}^{\mathrm{ns}}$	67.4 ± ${1.3\;}_{\mathrm{NS}}^{\mathrm{ns}}$	54.4 ± ${0.6\;}_{\mathrm{NS}}^{\mathrm{ns}}$	2.0 ± ${0.2\;}_{\mathrm{NS}}^{\mathrm{ns}}$		10	41.4 ± ${1.1\;}_{\mathrm{NS}}^{\mathrm{ns}}$	63.0 ± ${1.7\;}_{\mathrm{NS}}^{\mathrm{ns}}$	52.0 ± ${1.4\;}_{\mathrm{NS}}^{\mathrm{ns}}$	1.9 ± ${0.5\;}_{\mathrm{NS}}^{\mathrm{ns}}$
SCLE.B	0	75.0 ± ${0.7\;}_{C}^{\mathrm{ns}}$	89.4 ± ${3.5\;}_{C}^{\mathrm{ns}}$	79.6 ± ${0.8\;}_{C}^{\mathrm{ns}}$	2.9 ± $\;0.7{\;}_{\mathrm{NS}}^{\mathrm{ns}}$	SCLE.Y	0	72.6 ± ${0.3\;}_{C}^{\mathrm{ns}}$	84.4 ± ${2.1\;}_{C}^{\mathrm{ns}}$	77.4 ± $0.6{\;}_{C}^{\alpha }$	2.9 ± ${0.4\;}_{\mathrm{NS}}^{\mathrm{ns}}$
	5	44.5 ± $\;0.3{\;}_{\mathrm{NS}}^{\mathrm{ns}}$	69.2 ± $\;2.3{\;}_{\mathrm{NS}}^{\mathrm{ns}}$	56.4 ± $\;0.4{\;}_{\mathrm{NS}}^{\mathrm{ns}}$	1.9 ±$\;0.4{\;}_{\mathrm{NS}}^{\mathrm{ns}}$		5	44.3 ± $\;1.6{\;}_{N.S}^{\mathrm{ns}}$	66.2 ± $\;1.0{\;}_{\mathrm{NS}}^{\mathrm{ns}}$	54.5 ± $\;0.9{\;}_{\mathrm{NS}}^{\mathrm{ns}}$	1.7 ± $1.3{\;}_{\mathrm{NS}}^{\mathrm{ns}}$
	10	44.0 ± $\;0.3{\;}_{\mathrm{NS}}^{\mathrm{ns}}$	68.3 ± $\;2.5{\;}_{\mathrm{NS}}^{\mathrm{ns}}$	55.0 ± $\;0.4{\;}_{\mathrm{NS}}^{\mathrm{ns}}$	1.9 ± $\;0.3{\;}_{\mathrm{NS}}^{\mathrm{ns}}$		10	42.3 ± $\;1.9{\;}_{\mathrm{NS}}^{\mathrm{ns}}$	60.4 ± $\;1.2{\;}_{\mathrm{NS}}^{\mathrm{ns}}$	52.0 ± $\;1.9{\;}_{\mathrm{NS}}^{\mathrm{ns}}$	0.73 ± $0.51{\;}_{\mathrm{NS}}^{\mathrm{ns}}$

Displayed data are expressed as mean values of three replications ± standard deviation. Values in the same line not sharing (i) lowercase superscript letters suggest significant differences (*p <* 0.05) amongst samples at a confidence level of 95%; ns: not significant differences amongst samples (*p >* 0.05); (ii) uppercase subscript letters suggest significant differences (*p <* 0.05) amongst samples treated with different raising conditions at a confidence level of 95% NS: Not significant treatment with different raising conditions (*p >* 0.05). C: Control Formulation; Y: Gluten-Free formulations with Dry Yeast; B: Gluten-free Formulations with Chemical Reagent Baking Powder; SCP: Sour-cherry Fruit Powder; SCLE: Sour-cherry Extract in Powder Form.

**Table 5:** Textural parameters of gluten-free doughs produced with or without supplementation with sour-cherry powder and lyophilized extract.

Samples	Hardness (N)	Cohesiveness	Gumminess	Springiness
Raising Agent: Baking Powder				
C.B	0.67 ± $0.12{\;}_{A}^{a}$	0.41 ± $0.01{\;}_{A}^{a}$	0.27 ± $0.05{\;}_{A}^{\mathrm{ns}}$	0.91 ± $0.11{\;}_{\mathrm{NS}}^{a}$
SCP.B	0.68 ± $0.11{\;}_{B}^{a}$	0.33 ± $0.01{\;}_{B}^{b}$	0.23 ± $0.05{\;}_{B}^{\mathrm{ns}}$	1.04 ± $0.03{\;}_{\mathrm{NS}}^{a,b}$
SCLE.B	1.08 ± $0.20{\;}_{C}^{b}$	0.28 ± $0.07{\;}_{C}^{b}$	0.28 ± $0.06{\;}_{C}^{\mathrm{ns}}$	1.19 ± $0.22{\;}_{\mathrm{NS}}^{b}$
Raising Agent: Dry Yeast				
C.Y	0.37 ± $0.06{\;}_{A}^{a}$	0.16 ± $0.01{\;}_{A}^{a}$	0.06 ± $0.01{\;}_{A}^{a}$	1.00 ± $0.00{\;}_{\mathrm{NS}}^{a}$
SCP.Y	0.48 ± $0.08{\;}_{B}^{a,b}$	0.20 ± $0.02{\;}_{B}^{b}$	0.10 ± $0.02{\;}_{B}^{a,b}$	1.00 ± $0.00{\;}_{\mathrm{NS}}^{a}$
SCLE.Y	0.65 ± $0.15{\;}_{C}^{b}$	0.19 ± $0.01{\;}_{C}^{b}$	0.11 ± $0.03{\;}_{C}^{b}$	1.17 ± $0.12{\;}_{\mathrm{NS}}^{b}$

Data are expressed as mean values of three replications ± standard deviation. Values in the same column not sharing (i) lowercase superscript letters suggest significant differences (*p <* 0.05) amongst samples at a confidence level of 95%; ns: not significant differences amongst samples (*p >* 0.05); (ii) uppercase subscript letters suggest significant differences (*p <* 0.05) amongst samples treated with different raising conditions at a confidence level of 95% NS: Not significant treatment with different raising conditions (*p >* 0.05). Abbreviations: C: Control Formulation; Y: Gluten-Free formulations with Dry Yeast; B: Gluten-free Formulations with Chemical Reagent Baking Powder; SCP: Sour-cherry Fruit Powder; SCLE: Sour-cherry Extract in Powder Form.

**Table 6:** Hardness and color parameter values of 3D-printed gluten-free snacks with or without supplementation with sour-cherry powder and lyophilized extract after the drying process.

Raising Agent: Baking Powder							
		Tray Drying			Vacuum Drying		
	**Samples**	**C.B.TD**	**SCP.B.TD**	**SCLE.B.TD**	**C.B.VD**	**SCP.B.VD**	**SCLE.B.VD**
Color parameters	L*	79.2 ± $0.9{\;}_{A}^{a}$	61.9 ± $0.4{\;}_{A}^{c}$	68.1 ± $0.1{\;}_{A}^{b}$	81.2 ± $0.2{\;}_{A}^{a}$	65.8 ± $0.0{\;}_{A}^{c}$	72.4 ± $0.1{\;}_{A}^{b}$
	a*	5.6± $0.4{\;}_{\mathrm{NS}}^{a}$	2.9 ± $0.1{\;}_{B}^{b}$	13.6 ± $0.1{\;}_{\mathrm{NS}}^{b}$	4.9 ± $0.1{\;}_{\mathrm{NS}}^{a}$	3.4 ± $0.0{\;}_{B}^{b}$	15.4 ± $0.0{\;}_{\mathrm{NS}}^{b}$
	b*	22.6 ± $0.4{\;}_{B}^{a}$	3.1 ± $0.0{\;}_{C}^{c}$	11.8 ±$\;0.1{\;}_{B}^{b}$	21.7 ± $0.1{\;}_{B}^{a}$	2.6 ± $0.2{\;}_{C}^{c}$	10.9 ± $0.0{\;}_{B}^{c}$
Texture	Hardness (N)	6.7 ± $0.2{\;}_{A}^{a}$	10.2 ±$1.8{\;}_{B}^{a,b}$	11.6 ± $2.6{\;}_{C}^{b}$	3.8 ± $0.5{\;}_{A}^{a,b}$	3.4 ± $0.8{\;}_{B}^{a}$	5.7 ± $0.7{\;}_{C}^{b}$
**Raising Agent: Dry Yeast**							
	**Samples**	**C.Y.TD**	**SCP.Y.TD**	**SCLE.Y.TD**	**C.Y.VD**	**SCP.Y.VD**	**SCLE.Y.VD**
Color parameters	L*	82.6 ± $0.1{\;}_{\mathrm{NS}}^{a}$	66.0 ± $0.2{\;}_{A}^{b}$	74.6 ±$\;0.4{\;}_{B}^{c}$	83.2 ± $1.0{\;}_{\mathrm{NS}}^{a}$	70.7 ± $0.1{\;}_{A}^{b}$	72.7 ± $0.4{\;}_{B}^{c}$
	a*	3.9 ± $0.1{\;}_{\mathrm{NS}}^{a}$	2.6 ± $0.0{\;}_{A}^{b}$	2.9 ± $0.1{\;}_{B}^{c}$	3.6 ± $0.3{\;}_{\mathrm{NS}}^{a}$	3.3 ± $0.0{\;}_{A}^{b}$	2.3 ± $0.1{\;}_{B}^{c}$
	b*	19.0 ± $0.6{\;}_{\mathrm{NS}}^{a}$	9.2 ± $0.1{\;}_{A}^{b}$	10.2 ± $0.1{\;}_{B}^{b}$	18.9 ± $0.4{\;}_{\mathrm{NS}}^{a}$	11.3 ± $0.1{\;}_{A}^{b}$	11.5 ± $0.1{\;}_{B}^{c}$
Texture	Hardness (N)	5.3 ± $0.21{\;}_{A}^{\mathrm{ns}}$	4.1 ± $0.8{\;}_{\mathrm{NS}}^{\mathrm{ns}}$	5.3 ± $0.8{\;}_{B}^{\mathrm{ns}}$	3.0 ± $0.2{\;}_{A}^{\mathrm{ns}}$	3.4 ± $0.8{\;}_{\mathrm{NS}}^{\mathrm{ns}}$	3.0 ± $0.1{\;}_{B}^{\mathrm{ns}}$

Displayed data are expressed as mean values of three replications ± standard deviation. Values in the same line not sharing (i) lowercase superscript letters suggest significant differences (*p <* 0.05) amongst samples at a confidence level of 95%; ns: not significant differences amongst samples (*p >* 0.05); (ii) uppercase subscript letters suggest significant differences (*p <* 0.05) amongst samples treated with different drying methods at a confidence level of 95% NS: Not significant treatment with different drying methods (*p >* 0.05). C: Control Formulation; Y: Gluten-free Formulations with Dry Yeast; B: Gluten-free Formulations with Chemical Reagent Baking Powder; SCP: Sour-cherry Fruit Powder; SCLE: Sour-cherry Extract in Powder Form; TD: Tray-Drying Process; VD: Vacuum-Drying Process.

## Data Availability

Not applicable.

## References

[1] Adem M., Sadik J., Worku A., Neela S. (2020). Optimization of lupine (*Lupinus albus* L.) composition, feed moisture content and barrel temperatures for best quality maize based extruded snack food. Nutr. Food Sci..

[2] Zhu S., Ramos V., Heckert O., Stieger M., van der Goot A., Schutyser M. (2022). Creating protein-rich snack foods using binder jet 3D printing. J. Food Eng..

[3] Agrahar-Murugkar D., Zaidi A., Dwiwedi S. (2018). Quality of nixtamalized, sprouted and baked multigrain chips. Nutr. Food Sci..

[4] Singh A., Kumari A., Chauhan A. (2022). Formulation and evaluation of novel functional snack bar with amaranth, rolled oat, and unripened banana peel powder. J. Food Sci. Technol..

[5] Alongi M., Anese M. (2021). Re-thinking functional food development through a holistic approach. J. Funct. Foods.

[6] Matos M.E., Rosell C.M. (2015). Understanding gluten-free dough for reaching breads with physical quality and nutritional balance. J. Sci. Food Agric..

[7] Ren Y., Linter B.R., Linforth R., Foster T.J. (2020). A comprehensive investigation of gluten free bread dough rheology, proving and baking performance and bread qualities by response surface design and principal component analysis. Food Funct..

[8] Arendt E.K., Bello D.F., Hamaker B.R. (2008). 19-Functional cereal products for those with gluten intolerance. Woodhead Publishing Series in Food Science, Technology and Nutrition, Technology of Functional Cereal Products.

[9] Cappelli A., Noemi O., Enrico C. (2020). A Systematic Review of Gluten-Free Dough and Bread: Dough Rheology, Bread Characteristics, and Improvement Strategies. Appl. Sci..

[10] Collar C., Conte P., Fadda C., Piga A. (2015). Gluten-free dough-making of specialty breads: Significance of blended starches, flours and additives on dough behaviour. Food Sci. Technol. Int..

[11] Megusar P., Stopar D., Ulrih N., Dogsa I., Prislan I. (2022). Thermal and Rheological Properties of Gluten-Free, Starch-Based Model Systems Modified by Hydrocolloids. Polym. J..

[12] El Khoury D., Balfour-Ducharme S., Joye I.J. (2018). A Review on the Gluten-Free Diet: Technological and Nutritional Challenges. Nutrients.

[13] Bonerz D., Würth K., Dietrich H., Will F. (2007). Analytical characterization and the impact of ageing on anthocyanin composition and degradation in juices from five sour cherry cultivars. Eur. Food Res. Technol..

[14] Cilek B., Luca A., Hasirci V., Sahin S., Sumnu G. (2012). Microencapsulation of phenolic compounds extracted from sour cherry pomace: Effect of formulation, ultrasonication time and core to coating ratio. Eur. Food Res. Technol..

[15] Nikolaou E.N., Karvela E.D., Marini E., Panagopoulou E.A., Chiou A., Karathanos V.T. (2022). Enrichment of bakery products with different formulations of bioactive microconstituents from black Corinthian grape: Impact on physicochemical and rheological properties in dough matrix and final product. J. Cereal Sci..

[16] Ferretti G., Bacchetti T., Belleggia A., Neri D. (2010). Cherry Antioxidants: From Farm to Table. Molecules.

[17] Wang H., Nair M.G., Strasburg G.M., Chang Y.-C., Booren A.M., Gray I., DeWitt D.L. (1999). Antioxidant and Antiinflammatory Activities of Anthocyanins and Their Aglycon, Cyanidin, from Tart Cherries. J. Nat. Prod..

[18] Blando F., Geraldi C., Nicoletti I. (2004). Sour Cherry (*Prunus cerasus* L.) Anthocyanins as Ingredients for Functional Foods. J. Biotechnol. Biomed..

[19] Wojdylo A., Nowicka P., Laskowski P., Oszmiański J. (2014). Evaluation of sour cherry (*Prunus cerasus* L.) fruits for their polyphenol content, antioxidant properties, and nutritional components. J. Agric. Food Chem..

[20] Petrović J., Pajin B., Lončarević I., Šaponjac V., Nikolić I., Ačkar Đ., Zarić D. (2019). Encapsulated sour cherry pomace extract: Effect on the colour and rheology of cookie dough. Food Sci. Technol. Int..

[21] Gumul D., Korus A., Ziobro R. (2020). Extruded Preparations with Sour Cherry Pomace Influence Quality and Increase the Level of Bioactive Components in Gluten-Free Breads. Int. J. Food Sci..

[22] Liu Z., Zhang M., Bhandari B., Wang Y. (2017). 3D printing: Printing precision and application in food sector. Trends Food Sci. Technol..

[23] Pulatsu E., Su J.-W., Lin J., Lin M. (2020). Factors affecting 3D printing and post-processing capacity of cookie dough. Innov. Food Sci. Emerg. Technol..

[24] Guo C., Zhang M., Devahastin S. (2021). Color/aroma changes of 3D-Printed buckwheat dough with yellow flesh peach as triggered by microwave heating of gelatin-gum Arabic complex coacervates. Food Hydrocoll..

[25] Pulatsu E., Lin M. (2021). A review on customizing edible food materials into 3D printable inks: Approaches and strategies. Trends Food Sci. Technol..

[26] Godoi F.C., Prakash S., Bhandari B.R. (2016). 3D printing technologies applied for food design: Status and prospects. J. Food Eng..

[27] Jiang H., Zheng L., Zou Y., Tong Z., Han S., Wang S. (2019). 3D food printing: Main components selection by considering rheological properties. Crit. Rev. Food Sci. Nutr..

[28] 28 Radoš K., Benković M., Mustač N., Habuš M., Voučko B., Pavičić T., Novotni D. (2023). Powder properties, rheology and 3D printing quality of gluten-free blends. J. Food Eng..

[29] Witczak M., Ziobro R., Juszczak L., Korus J. (2016). Starch and starch derivatives in gluten-free systems e a review. J. Cereal Sci..

[30] Matas A., Igual M., García-Segovia P., Martínez-Monzó J. (2022). Application of 3D Printing in the Design of Functional Gluten-Free Doug. Foods.

[31] Wang M., Li D., Zang Z., Sun X., Tan H., Si X., Liu R. (2022). 3D food printing: Applications of plant-based materials in extrusion-based food printing. Crit. Rev. Food Sci. Nutr..

[32] Sun J., Zhou W., Yan L., Huang D., Lin L.-Y. (2018). Extrusion-based food printing for digitalized food design and nutrition control. J. Food Eng..

[33] Kim H., Lee I., Park S., Lee J., Nguyen M.-H., Park H. (2019). Effect of hydrocolloid addition on dimensional stability in post-processing of 3D printable cookie dough. LWT.

[34] Zhang L., Lou Y., Schutyser M. (2018). 3D printing of cereal-based food structures containing probiotic. Food Struct..

[35] Conte P., Del Caro A., Urgeghe P., Petretto G., Montanari L., Piga A., Fadda C. (2020). Nutritional and aroma improvement of gluten-free bread: Is bee pollen effective?. LWT.

[36] Arnous A., Makris D.P., Kefalas P. (2002). Correlation of Pigment and Flavanol Content with Antioxidant Properties in Selected Aged Regional Wines from Greece. J. Food Compos. Anal..

[37] Yilmaz Y., Akgun F. (2008). Ferric reducing/antioxidant power of Maillard reaction products in model bread crusts. J. Food Agric. Environ..

[38] Witczak T., Juszczak L., Ziobro R., Korus J. (2017). Rheology of gluten-free dough and physical characteristics of bread with potato protein. J. Food Process Eng..

[39] Korus J., Juszczak L., Witczak M., Ziobro R. (2020). Effect of Citrus Fiber on the Rheological Properties of Dough and Quality of the Gluten-Free Bread. Appl. Sci..

[40] Witczak M., Juszczak L., Ziobro R., Korus J. (2012). Part I: Rheological and thermal properties of gluten-free dough. Food Hydrocoll..

[41] Ronda F., Villanueva M., Collar C. (2014). Influence of acidification on dough viscoelasticity of gluten-free rice starch-based dough matrices enriched with exogenous protein. LWT-Food Sci. Technol..

[42] Horstman S., Axel C., Arendt E. (2018). Water absorption as a prediction tool for the application of hydrocolloids in potato starch-based bread. Food Hydrocoll..

[43] Ngo T., Kusumawardani S., Kusumawardani K., Luangsakul N. (2022). Polyphenol-Modified Starches and Their Applications in the Food Industry: Recent Updates and Future Directions. Foods.

[44] Zhu F., Cai Y.-Z., Sun M., Corke H. (2009). Effect of phytochemical extracts on the pasting, thermal, and gelling properties of wheat starch. Food Chem..

[45] Khoo H., Azian A., Tang S., Lim S. (2017). Anthocyanidins and anthocyanins: Colored pigments as food, pharmaceutical ingredients, and the potential health benefits. J. Food Nutr. Res..

[46] Šaponjac V., Ćetković G., Čanadanović-Brunet J., Pajin B., Djilas S., Petrović J., Vulić J. (2016). Sour cherry pomace extract encapsulated in whey and soy proteins: Incorporation in cookies. Food Chem..

[47] Nunes M.A., Reszczyński F., Páscoa R.N.M.J., Costa A.S.G., Alves R.C., Oliveira M.B.P.P. (2021). Influence of Olive Pomace Blending on Antioxidant Activity: Additive, Synergistic, and Antagonistic Effects. Molecules.

[48] Durand E., Zhao Y., Coupland J.N., Elias R.J. (2015). Assessing interactions between lipophilic and hydrophilic antioxidants in food emulsions. J. Agric. Food Chem..

[49] Bolling B.W., Chen Y.Y., Chen C.Y. (2013). Contributions of phenolics and added vitamin C to the antioxidant capacity of pomegranate and grape juices: Synergism and antagonism among constituents. Int. J. Food Sci. Technol..

